# Needle-free connectors in tunneled central venous catheters for hemodialysis: A prospective single-centre safety and feasibility study

**DOI:** 10.3205/dgkh000616

**Published:** 2026-01-16

**Authors:** Boštjan Leskovar, Tjaša Furlan

**Affiliations:** 1 Vascular Disease and Vascular Access Unit, Trbovlje General Hospital, Trbovlje, Slovenia

**Keywords:** hemodialysis, tunnelled central venous catheter, needle-free connector, catheter-related infection

## Abstract

**Background:**

Tunnelled central venous catheters (t-CVCs) remain essential for hemodialysis when arteriovenous access is not feasible, but catheter-related bloodstream infection (CRBSI) is a major risk. In temporary CVCs, needle-free connectors have been associated with fewer infections; however, data on high-flow hemodialysis catheters are limited. We evaluated the short-term safety and feasibility of high-flow split-septum needle-free connectors on hemodialysis temporary central venous catheters (t-CVCs).

**Methods:**

In a prospective single-centre study (Trbovlje General Hospital, Slovenia; June 2025), adults with a mature t-CVC used for thrice-weekly hemodialysis and without signs of infection were enrolled. Split-septum needle-free connectors (Asset-FlowArt^®^-1010H-S) with antibacterial caps were applied to both hubs and used for 12 consecutive dialysis sessions per patient. The primary outcome was microbiological safety, defined as negativity of paired blood cultures after 12 sessions. Secondary outcomes included clinical tunnel infection, change in inflammatory markers, need to modify dialysis prescription/anticoagulation/catheter care, mechanical complications, hospitalization, and death.

**Results:**

Fifteen patients completed 12 sessions each (total 180 sessions). Paired blood cultures were negative at baseline and after 12 sessions in all patients. No tunnel infections occurred. There were no meaningful changes in inflammatory markers, and no device-related adverse events or protocol modifications were required. No hospitalizations or deaths occurred during follow-up. Flow performance supported prescribed blood-flow rates.

**Conclusion:**

In this short-term single-centre cohort, high-flow split-septum needle-free connectors appeared feasible and microbiologically safe on hemodialysis t-CVCs, without compromising dialysis delivery or routine catheter care. Larger, multicenter randomized trials with longer follow-up are warranted to determine effects on CRBSI incidence and catheter patency.

## Zusammenfassung

**Hintergrund:**

Getunnelte zentralvenöse Katheter (t-ZVK) sind für die Hämodialyse unverzichtbar, wenn eine arteriovenöse Fistel/Prothese nicht möglich ist; jedoch bleibt die Katheter-assoziierte Blutstrominfektion (CRBSI) ein zentrales Risiko. Bei temporären ZVKs wurden mit nadelfreien Konnektoren geringere Infektionsraten beobachtet. Die Evidenz für hochflussfähige Hämodialysekatheter ist jedoch begrenzt. Ziel war die Bewertung der kurzfristigen Sicherheit und Machbarkeit von hochflussfähigen Split-Septum-nadelfreien Konnektoren an t-ZVKs.

**Methode:**

In einer prospektiven, monozentrischen Studie (Allgemeinkrankenhaus Trbovlje, Slowenien; Juni 2025) wurden erwachsene Hämodialysepatient:innen mit maturem t-ZVK, der für die dreimal wöchentliche Hämodialyse verwendet wurde und keine Anzeichen einer Infektion aufwies, aufgenommen. Split-Septum-nadelfreie Konnektoren (Asset-FlowArt^®^-1010H-S) mit antibakteriellen Kappen wurden an beiden Hubs angebracht und für 12 aufeinanderfolgende Dialysesitzungen pro Person verwendet. Der primäre Endpunkt war die mikrobiologische Sicherheit (Negativität gepaarter Blutkulturen nach 12 Sitzungen). Sekundäre Endpunkte umfassten klinische Tunnelinfektion, Entzündungsmarker, Bedarf an Änderungen der Dialyse/Antikoagulation/Pflege, mechanische Komplikationen, Hospitalisation und Tod.

**Ergebnisse:**

15 Patient:innen absolvierten jeweils 12 Sitzungen (insgesamt 180). Gepaarte Blutkulturen waren zu Beginn und nach 12 Sitzungen bei allen negativ. Es traten keine Tunnelinfektionen auf. Entzündungsmarker zeigten keine relevanten Veränderungen; es waren keine Protokollanpassungen oder Geräte-assoziierten unerwünschten Ereignisse erforderlich. Hospitalisationen oder Todesfälle traten nicht auf. Die Flussleistung erlaubte die verordneten Blutflüsse.

**Schlussfolgerung:**

In dieser kurzzeitigen, monozentrischen Kohorte erwiesen sich hochflussfähige Split-Septum-nadelfreie Konnektoren an Hämodialyse-t-ZVKs als machbar und mikrobiologisch sicher, ohne die Dialysedurchführung oder die Routinepflege zu beeinträchtigen. Größere, multizentrische, randomisierte Studien mit längerer Nachbeobachtung sind erforderlich, um Effekte auf CRBSI-Inzidenz und Katheterpatenz zu klären.

## Introduction

Tunnelled central venous catheters (t-CVCs) are an essential vascular access for hemodialysis when a functional arteriovenous fistula or graft is unavailable or not feasible. They may provide short- to intermediate-term access or serve as a definitive solution in patients with limited alternatives [1], [2]. Current NKF-KDOQI guidance recommends a tunnelled device whenever a so-called temporary catheter is anticipated to be required for more than three weeks.

Infection is the complication of most tremendous significance, driving excess morbidity, hospitalizations, and mortality among hemodialysis patients [3]. In t-CVCs, catheter-related infections include catheter-related bloodstream infection (CRBSI) and infections of the subcutaneous tunnel. The reported incidence of hemodialysis catheter-related bloodstream infections in the literature is approximately 0.35 to 0.73/1,000 catheter days [4], [5], [6].

Multiple strategies aim to reduce CRBSI risk – among them antimicrobial lock solutions [7] and coordinated prevention bundles that standardize hub disinfection, hand hygiene, and connection procedures [8], [9]. Equally important are clear, consistent nursing instructions and patient education at discharge to maintain safe catheter care across settings.

In temporary CVCs, the introduction of needle-free connectors has been associated with lower catheter-related infection [10], [11] and occlusion rates [12]. In Germany, the Commission for Hospital Hygiene and Infection Prevention (KRINKO) at the Robert Koch Institute recommends the use of needle-free connectors to reduce the risk of CRBSI [13]. This recommendation is based on the premise that needle-free connectors may facilitate the handling of central venous catheter connections, thereby reducing the window for microbial contamination of the ports.

The broader adoption of such connectors on hemodialysis t-CVCs has been limited historically by the need for blood-flow rates exceeding 300 mL/min and by earlier designs that utilized intraluminal springs – features that posed challenges for both adequate flow and long-term microbiological neutrality. Newer silicone split-septum designs overcome these constraints, enabling high-flow use [14]. 

We aimed to assess the short-term safety and effectiveness of needle-free connectors in preventing CRBSI in t-CVCs. 

## Methods

### Study design

We conducted a prospective single-centre study at the hemodialysis centre in Trbovlje General Hospital, Trbovlje, Slovenia. In June 2025, we included all hemodialysis patients at our centre with a t-CVC who fulfilled the inclusion criteria and exclusion criteria. The indication for a t-CVC insertion in patients with end-stage kidney disease was a reduced left ventricular ejection fraction (<30%), polymorbidity with a short life expectancy (<1 year), or a condition of the vascular system where arteriovenous fistula or graft construction was not possible. 

In all included patients, we used needle-free connectors on t-CVCs as per the study protocol.

### Inclusion and exclusion criteria

Inclusion criteria for the study were:

- age >18 years,
- a t-CVC inserted >than 30 days ago,
- regular hemodialysis three times weekly using the t-CVC,
- mature subcutaneous tunnel with an ingrown catheter cuff,
- no signs of local or systemic infection at the time of inclusion,
- signed informed consent.

Exclusion criteria for the study were:

- pregnancy,
- signs of mechanical damage to the t-CVC,
- predicted survival of less than 3 months (patients on palliative care for cancer, neurologic disease, cardiovascular disease).

### Study protocol

All patients who fulfilled the inclusion and exclusion criteria were included in the study. The index date was the date the informed consent was signed. On the index day, following standard sterile t-CVC care, paired blood cultures were drawn from each lumen of the t-CVC, and blood tests were performed (C-reactive protein (CRP), procalcitonin (PCT), and complete blood count with differential). High-flow split-septum needle-free connectors (Asset-FlowArt 1010H-S) were installed on both Luer-Lock hubs and covered with antibacterial caps, in accordance with the standard practice at our institution. Needle-free connectors were used with t-CVCs for six consecutive hemodialysis sessions, then the needle-free connectors were replaced and used for another six sessions (total 12 sessions per patient). Before the 13^th^ session, blood tests (including cultures, CRP, PCT, and a complete blood count with differential) were repeated. Throughout the study, dialysis prescriptions and intradialytic anticoagulation were not modified. Catheter care was performed in accordance with our long-standing aseptic protocol: after removing the antibacterial cap, a compatible disinfectant was applied to the needle-free connector. The lumens were flushed with at least 20 mL of 0.9% NaCl, and dialysis lines were connected to both arterial and venous limbs. Post-dialysis, each lumen was flushed with ≥20 mL of 0.9% NaCl and locked with 30% sodium citrate in a volume of 0.1 mL above the manufacturer’s listed lumen volume for two to five sessions consecutively. Every third to sixth session, alteplase was used as a lock solution to support long-term patency and disrupt intraluminal biofilm [15], [16].

### Outcomes

The primary outcome was microbiological safety, as indicated by the negativity of paired blood cultures after 12 dialysis sessions (3 sessions per week, totaling 1 month of observation) using needle-free connectors. Secondary outcomes included clinical signs of tunnel infection, changes in CRP, PCT, leukocyte count, and differential blood count; the need to modify dialysis prescription, anticoagulation, or catheter-care regimen; and any adverse events (mechanical damage to the t-CVC, patient hospitalization, or death).

### Statistical analysis

Statistical analyses were performed using Stata 17.0 for Mac (StataCorp LLC, 2017). Continuous data were summarised as means (with standard deviation) for normally distributed variables and medians (with interquartile range) for non-normally distributed variables. Categorical data were summarized as counts and percentages. Within-patient changes from the index date to the end of the study were assessed using paired t-tests or signed-rank test as appropriate. Statistical significance was set at two-tailed *p*<0.05.

### Ethics and data availability

The National Medical Ethics Committee of the Republic of Slovenia approved the study (Approval No. KME-0120-446/2024-2711-7). All participants provided written informed consent. De-identified data and the analysis code will be made available upon reasonable request or deposited in a public repository upon acceptance.

## Results

### Study population

**Figure 1:**
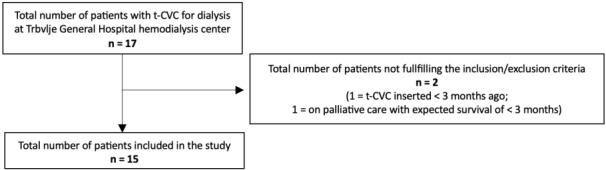
The CONSORT diagram for the study

In June 2025, 17 patients had a t-CVC as their vascular access at our hemodialysis centre, of which 15 patients were included in the study (Figure 1 (Fig. 1)). All patients had an Arrow-VectorFlow^®^ t-CVC inserted through the left or right jugular vein. Patients’ demographics are presented in Table 1 (Tab. 1).

### Study outcomes

All 15 patients had negative paired blood cultures at both baseline and after 12 dialysis sessions using needle-free connectors, for a total of 180 dialysis procedures, without microbiological evidence of catheter-related infection. No tunnel infections or significant difference in biochemical parameters were observed. Dialysis performance was adequate for the prescribed blood-flow rates, and no other adverse events were recorded that would require alteration of the dialysis protocol, anticoagulation, or catheter-care routine. There were no hospitalizations or deaths from any cause during the study. Outcomes are presented in Table 2 (Tab. 2).

In two patients the value of CRP and PCT increased during the study (CRP 10→87 mg/l, PCT 0.22→2.2 ng/ml; and CRP 9→153 mg/l, PCT 0.23→2.58 ng/ml) but both suffered infection of another origin at the end of the study, not related to t-CVC (one patient has diabetic foot infection, the other pneumonia).

## Discussion

In this short-term, prospective, single-centre evaluation of t-CVCs, the introduction of high-flow, split-septum needle-free connectors was not associated with microbiological evidence of CRBSI and did not worsen inflammatory markers. Dialysis delivery and routine catheter-care workflows were preserved without protocol alterations. Although modest in scale, these findings support the feasibility of integrating modern split-septum needle-free connectors into hemodialysis practice when accompanied by standardized hub disinfection and established lock regimens.

Prior studies in temporary CVCs have linked the use of needle-free connectors to lower infection rates when implemented alongside robust asepsis and staff training [10], [11]. Historically, the adoption of hemodialysis t-CVCs lagged due to concerns about achievable blood-flow rates (greater than 300 mL/min) and the microbiological implications of internal mechanical components. The split-septum design addresses both issues by offering a straight, cleanable fluid path and a sufficient cross-section for high flows [14]. Our results support this rationale: across 180 dialysis procedures, no positive blood cultures or clinical signs of tunnel infections were observed, and no adjustments to anticoagulation, lock solutions, or nursing protocols were required. From a pathophysiological standpoint, reducing manipulation at the Luer-Lock interface and providing continuous passive antisepsis between sessions (via antibacterial caps) plausibly limits hub contamination and intraluminal biofilm formation – key steps in CRBSI pathogenesis.

For units seeking incremental risk reduction without significant workflow changes, a split-septum needle-free plus antibacterial cap strategy may:

- standardize a closed, disinfectable connection point;
- add a visible compliance cue (cap in place) between treatments; and
- provide an additional safety barrier should a clamp be inadvertently opened or a cap become dislodged, potentially mitigating bleeding risks. Importantly, we observed no degradation in delivered dialysis (subjectively adequate blood-flow rates and no increase in alarms) and no signal toward greater thrombotic occlusion – outcomes that matter for day-to-day usability and patient comfort.

These feasibility data support a larger, adequately powered, multicenter randomized trial with at least 12 months of follow-up. Key design elements should include:

- patient-level randomization to needle-free connectors versus standard hubs (both arms embedded within a common infection-prevention bundle);
- standardized definitions and adjudication of CRBSI, exit-site, and tunnel infections;
- prespecified primary endpoint of CRBSI incidence per 1,000 catheter-days with time-to-first event analysis; and
- secondary endpoints covering catheter patency, thrombotic occlusion, delivered blood-flow rate and Kt/V, hospitalization, access removal/exchange, mortality, patient-reported experience, and cost-effectiveness.

## Limitations

Several limitations temper inference. First, the sample size was small and the observation window brief; with zero events, precision is limited, and we cannot estimate incidence or demonstrate statistical non-inferiority relative to standard practice. Second, the single-centre design may limit generalizability to settings with different patient case-mixes or catheter-care protocols. Finally, the study was not randomized, blinded, or monitored for adherence beyond routine supervision, leaving room for selection bias. 

Nevertheless, within the constraints of a small, single-centre study, high-flow split-septum needle-free connectors appeared feasible, safe, and compatible with routine hemodialysis workflows over the course of four weeks. While the absence of infections is encouraging, definitive conclusions about effectiveness require larger trials with longer follow-up and rigorous outcome assessment.

## Conclusions

High-flow split-septum needle-free connectors on tunneled hemodialysis catheters were feasible and appeared microbiologically safe over 12 consecutive dialysis procedures in a single-centre cohort. Further adequately powered randomized trials are warranted.

## Notes

### Authors’ ORCIDs

- Leskovar B: https://orcid.org/0000-0002-1100-1805
- Furlan T: https://orcid.org/0000-0003-0627-2050

### Funding

The authors received no funding for this study.

### Acknowledgments

The authors thank the Department of Haemodialysis for their cooperation, desire to learn, and striving for progress.

### Competing interests

Biomedis M.B. d.o.o. provided Asset-FlowArt^®^-1010H-S needle-free connectors free of charge for this study. Asset Inc. or Biomedis did not influence or participate in patient selection, data acquisition, data analysis or the writing of the manuscript.

## Figures and Tables

**Table 1:**
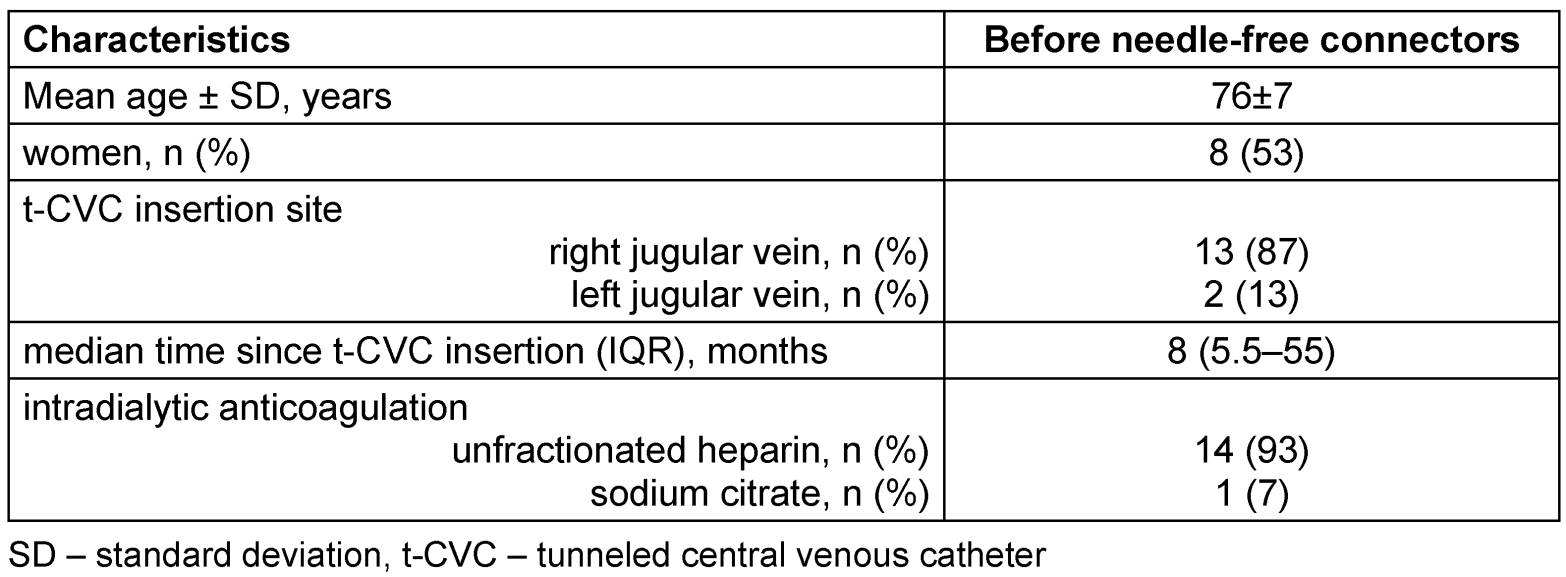
Patients’ demographics

**Table 2:**
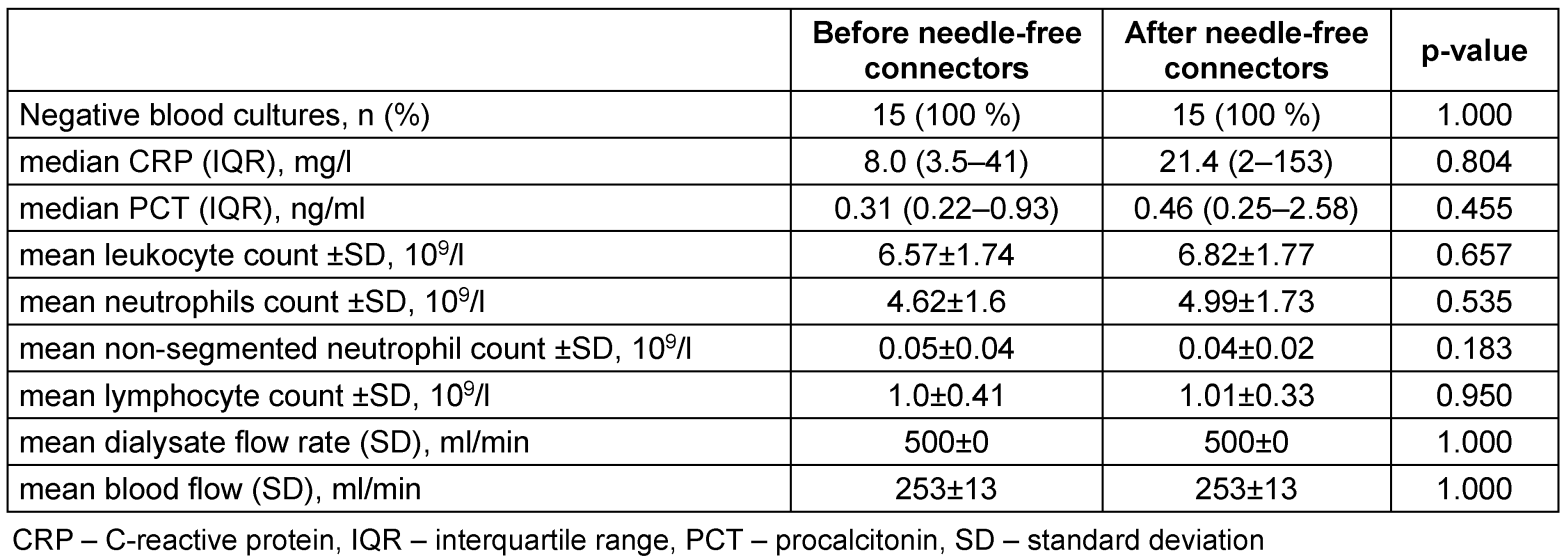
Primary and secondary study outcomes
